# P-853. Prevalence, Clinical Data and Associated Risk Factors of Third Generation Cephalosporin Resistance in *Salmonella* spp. Septicemia in Northeast of Thailand

**DOI:** 10.1093/ofid/ofae631.1045

**Published:** 2025-01-29

**Authors:** Kunakorn Thivakorakot, Atibordee Meesing, Supranee Phanthanawiboon

**Affiliations:** Faculty of medicine, Khon Kaen University, Khon Kaen, Khon Kaen, Thailand; Khon Kaen university, A. Muang, Khon Kaen, Thailand; Faculty of Medicine, Khon Kaen University, Khon Kaen, Khon Kaen, Thailand

## Abstract

**Background:**

The increase of antimicrobial resistance bacteria among foodborne pathogens including *Salmonella* spp. has been associated with poor outcomes. The studies of prevalence and risk factors of *Salmonella* spp. resistance in Thailand is limited. Hence, this study was conducted to study the prevalence and risk factors of third-generation cephalosporin resistance in *Salmonella* septicemic patients.

Heat map showing the proportion of antimicrobial resistance in Salmonella spp.

**Figure:**
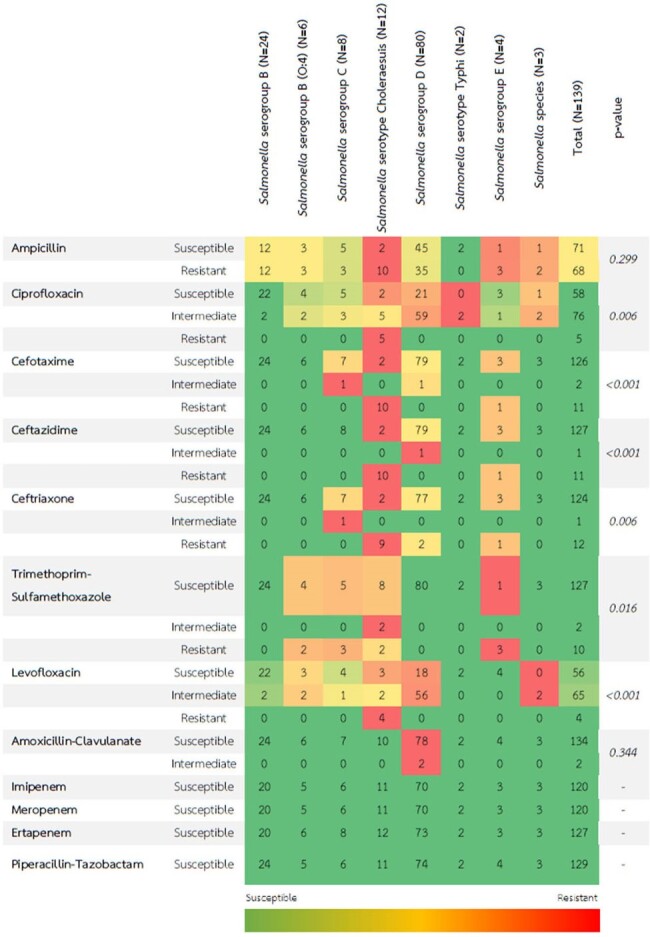

**Methods:**

A retrospective study was conducted between January 1st, 2016, to June 30th, 2023, in Srinagarind Hospital Khon Kaen, Thailand. All patients with hemoculture positive for *Salmonella* spp. who aged ≥ 18 years were included for analysis.

Subgroup analysis

**Figure:**
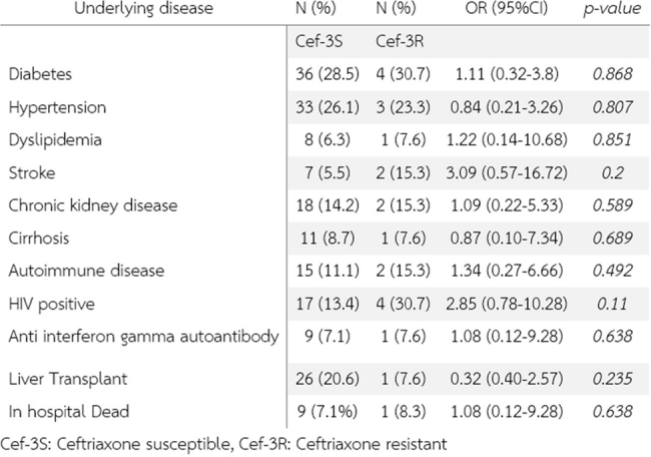

**Results:**

A total of 139 patients with Salmonella septicemia were included in this study, The mean age was 56.54 years and males were 51.8%. The prevalence of third-generation cephalosporin resistance *Salmonella* spp. was 8.8%. The most common primary infection site was primary bacteremia (66.9%), gastrointestinal tract (12.9%), Urinary tract (7.2%), bone and joint (3.6%), and arteritis (3.6%) respectively. Mortality was 7.1% (1 patient died from resistant strain). Carbapenem, piperacillin/tazobactam, and ciprofloxacin were an alternative treatment in the resistant group. There are no statistically significant among previously known factors associated with third-generation cephalosporin resistance in *Salmonella* septicemic patients.

Vital signs and laboratory investigations

**Figure:**
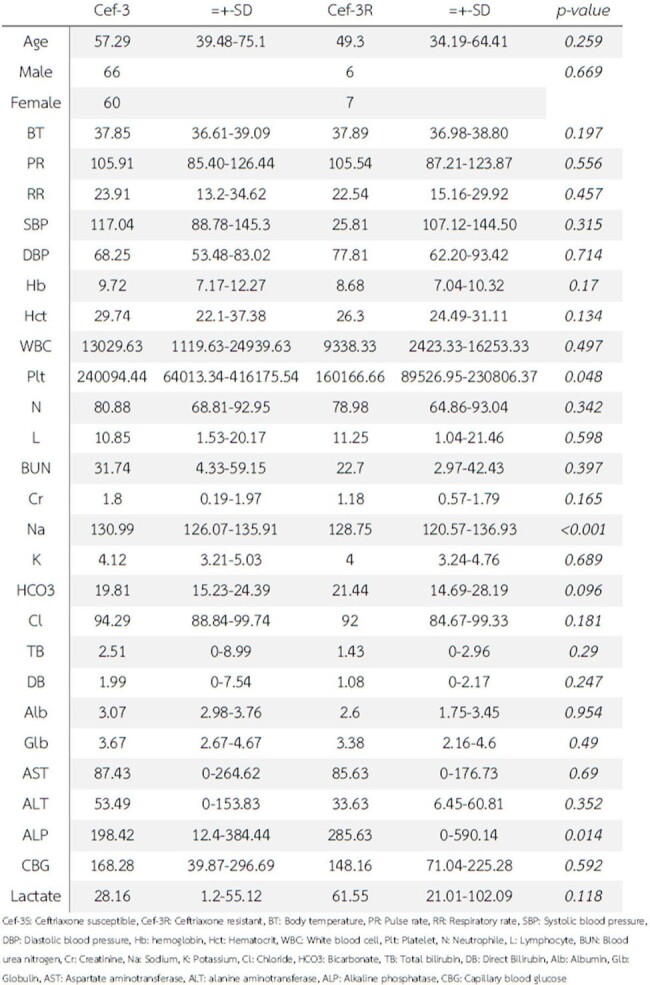

**Conclusion:**

The prevalence of third-generation cephalosporin resistance in *Salmonella* septicemic patients in this study was higher than overall in Thailand but lower than in tertiary care hospitals in Bangkok. Mortality was not higher in the resistant group. The mortality rate is not associated with ceftriaxone resistance in *Salmonella* spp. septicemia.

Empirical antibiotic of choice

**Figure:**
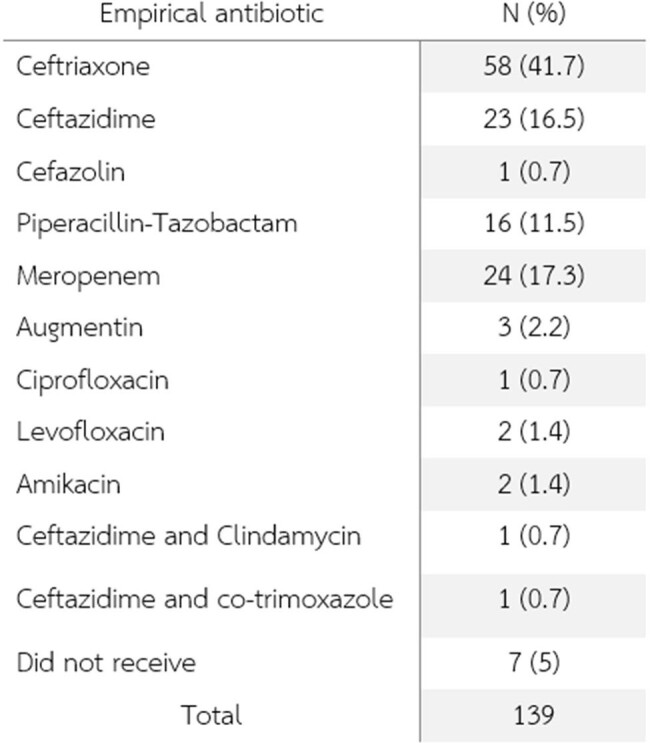

**Disclosures:**

**All Authors**: No reported disclosures

